# Artificial Hand

**Published:** 1855-06

**Authors:** 


*Artificial Hand.*—We have seen at the office of a friend in New York, an artificial hand, which possessed unusual merit. By a very ingenious mechanism the fingers were made to bend at each of the three joints together, as in the living hand. The hand examined was intended for a gentleman who had lost all save the fore-finger of the right hand. By a simple contrivance the three artificial fingers were made to follow accurately the motions of the index finger, and the limb was a very useful one indeed. We may confer a favor on some of our readers by giving the name of the artizan. He is a German named Faber, residing at No. 544 8th Avenue, New York, between 39th and 40th streets.

From the specimen of his workmanship mentioned above, we have no hesitation in recommending him.

